# N^ε^-Carboxymethyl Modification of Lysine Residues in Pathogenic Prion Isoforms

**DOI:** 10.1007/s12035-015-9200-8

**Published:** 2015-05-16

**Authors:** Yeong-Gon Choi, Hae-Young Shin, Jae-Il Kim, Eun-Kyoung Choi, Richard I. Carp, Yong-Sun Kim

**Affiliations:** 1 Ilsong Institute of Life Science, Hallym University, 1605-4 Gwanyangdong, Dongan-gu, Anyang, Gyeonggi-do 431-060 South Korea; 2 Department of Food Science and Nutrition, Bukyong National University, Busan, South Korea; 3 New York State Institute for Basic Research in Developmental Disabilities, Staten Island, NY 10314 USA; 4 Department of Microbiology, College of Medicine, Hallym University, 1 Hallymdaehak-gil, Chuncheon, Gangwon-do 200-702 South Korea

**Keywords:** Prion disease, Prions, 263K, Advanced glycation end products, N^ε^-(carboxymethyl)lysine

## Abstract

The most prominent hallmark of prion diseases is prion protein conversion and the subsequent deposition of the altered prions, PrP^Sc^, at the pathological sites of affected individuals, particularly in the brain. A previous study has demonstrated that the N-terminus of the pathogenic prion isoform (PrP^Sc^) is modified with advanced glycation end products (AGEs), most likely at one or more of the three Lys residues (positions 23, 24, and 27) in the N-terminus (23KKRPKP28). The current study investigated whether N^ε^-(carboxymethyl)lysine (CML), a major AGE form specific to Lys residues produced by nonenzymatic glycation, is an AGE adduct of the N-terminus of PrP^Sc^. We show that CML is linked to at least one Lys residue at the N-terminus of PrP^Sc^ in 263K prion-infected hamster brains and at least one of the eight Lys residues (positions 101, 104, 106, 110, 185, 194, 204, and 220) in the proteinase K (PK)-resistant core region of PrP^Sc^. The nonenzymatic glycation of the Lys residue(s) of PrP^Sc^ with CML likely occurs in the widespread prion-deposit areas within infected brains, particularly in some of the numerous tyrosine hydroxylase-positive thalamic and hypothalamic nuclei. CML glycation does not occur in PrP^C^ but is seen in the pathologic PrP^Sc^ isoform. Furthermore, the modification of PrP^Sc^ with CML may be closely involved in prion propagation and deposition in pathological brain areas.

**Electronic supplementary material:**

The online version of this article (doi:10.1007/s12035-015-9200-8) contains supplementary material, which is available to authorized users.

## Introduction

Prions, which are likely primarily composed of the pathogenic prion isoform (PrP^Sc^), are deposited in pathological areas, particularly in the brains of prion diseases that occur in humans and animals [1]. Prions induce the disease by converting the cellular prion isoform PrP^C^ into PrP^Sc^, which occurs in an enigmatic manner that remains elusive [2]. PrP^C^ undergoes a series of posttranslational and conformational change(s) which probably contribute in unknown way(s) to the conversion of PrP^C^ (in the presence of PrP^Sc^) into PrP^Sc^. The mechanism that converts the cellular prion isoform into the pathogenic isoform must be elucidated to ultimately develop efficient therapeutics for these diseases [3]. Despite the numerous attempts to determine the structure of PrP^Sc^ as the first step to describe the chain of prion conversion reactions, its definitive structure remains unclear [3]. The structure of PrP^C^ has been determined, whereas that of PrP^Sc^ has merely been theoretically proposed using methods such as Fourier transform infrared spectroscopy, circular dichroism spectroscopy, electron microscopy and crystallography, nuclear magnetic resonance, X-ray fiber diffraction, small angle X-ray scattering, hydrogen/deuterium exchange, limited proteolysis by proteinase K (PK), and antibody application to linearly or discontinuously probe the small surface accessible and secondary structural segments of the PrP protein backbone [3–18]. The ad interim trials seeking to reveal the definitive structure of PrP^Sc^ have not been productive, and the lack of an experimentally determined PrP^Sc^ structure is likely due to the structural variants of PrP^Sc^ (PrP^Sc^ heterodimers and additional multimers) that undergo a variety of stoichiometric conformational transformation(s) in prion-infected individuals [3, 19].

Advanced glycation end products (AGEs) are produced by the nonenzymatic glycation of amino compounds (i.e., proteins) with reducing sugars through a series of sequential and irreversible reactions, which exhibit some reversibility during the early stage of the reaction [20]. Various AGEs can be generated in vitro through the nonenzymatic glycation between bovine serum albumin (BSA) as a source of amino compounds and glucose (or glucose-6-phosphate, glyoxal, methylglyoxal, glycoaldehyde, or 3-deoxyglucosone) as one of the carbohydrates; these AGEs have been used as specific immunogens to produce anti-AGE(s) antibodies with a variety of anti-AGEs IgG populations or a specific anti-AGE IgG population [20–24]. Certain types of AGEs are deposited in in vivo pathological areas associated with numerous diseases featuring abnormal circulatory metabolism such as diabetes, hypertension, and atherosclerosis, thereby exacerbating these disorders. AGEs are also deposited in the hippocampal CA4 pyramidal neurons within elderly human brains, a finding that suggests their involvement in the aging process [20, 24–26].

A previous study showed that at least one Lys residue at the N-terminus of PrP^Sc^ of 263K prion-infected hamsters was linked with AGEs [27]. In addition, the pathogenic prion isoform PrP^Sc^ was modified with AGEs in the brains of 139H prion-infected hamsters, as well as in ME7-, 22L-, 139A-, and 87V-prion-infected mice and in human patients with sporadic Creutzfeldt–Jakob disease (CJD) or variant CJD. The fact that PrP^C^ was not glycated in these conditions suggests that the pathogenic prion isoform becomes nonenzymatically glycated with any form of carbohydrate in the brains of the prion-affected individuals during the long incubation period [27]. The current study investigated whether the N-terminal AGE is CML, which is a major type of AGE that modifies Lys residues [20]. The N-terminal AGEs of pathogenic prion isoforms have been affinity-isolated using 3F4 anti-PrP IgG and the R3 anti-AGEs antibody developed in previous studies [27, 28]. Using these reagents, we identified CML as an N-terminal AGE of pathogenic prion isoforms. Although the functional role of the CML linkage to pathogenic prion isoforms is currently unknown, it might be closely related to prion propagation.

## Materials and Methods

### Antibodies and Reagents

For PrP, the 3F4 and 3F10 anti-PrP IgGs (mouse monoclonal) [28, 29] and the 78295 anti-PrP antibody (rabbit polyclonal) [30] were used in this study. For CML, NF-1G and CMS-10 anti-CML IgGs (mouse monoclonal, Cosmo Bio Co., Ltd., Japan) [23] were used; for AGEs, the R3 anti-AGEs antibody (rabbit polyclonal) [27] was used; for tyrosine hydroxylase (TH), anti-TH IgG (mouse monoclonal, Santa Cruz Biotechnology, USA) was used; and for β-actin, anti-β-actin IgG (mouse monoclonal, Sigma, USA) was used. All other reagents not described were purchased from Sigma.

### Animal and Prion Strain

The hamster-adapted 263K prion strain was kindly provided by Richard H. Kimberlin (MRC Neuropathogenesis Unit, UK). Six-week-old male golden Syrian hamsters (*n* = 20, ORIENTBIO, Korea) were intracerebrally inoculated with 1 % (*w*/*v*) brain homogenates (50 μl) prepared in 0.01 M phosphate-buffered saline (PBS, pH 7.4) from either normal hamster brain or hamster-adapted 263K prion-infected brain at the terminal stage of the disease. The brains were harvested after the clinical signs were evident (70 days post-inoculation, dpi). The “Principles of laboratory animal care” (NIH publication no. 86-23, revised 1985) was followed, as well as a specific national law of the Republic of Korea on the protection of experimental animals. The Hallym University Animal Experimentation Committee approved all of the animal protocols used in this study.

### Immunofluorescence

After the hamsters were anesthetized at the terminal stage of the disease (70 dpi), they were transcardially perfused with a cold 0.05 M sodium phosphate buffer and then fixed with cold 4 % paraformaldehyde in 0.05 M sodium phosphate buffer. The hamster brains were immediately removed and then postfixed in 4 % paraformaldehyde for 12 h at 4 °C, rinsed with 0.05 M sodium phosphate buffer, dehydrated with sucrose for 12 h at 4 °C, and then cryo-cut using a microtome into 25-μm-thick coronal sections. After treatment with an avidin/biotin blocking kit (Vector Laboratories, USA), the sections were incubated with primary antibodies and sequentially with a biotinylated anti-mouse IgG, followed by treatment with Fluorescein Avidin DCS (Vector Laboratories, USA) for 3F4, NF-1G, or CMS-10. The sections were re-treated with an avidin/biotin blocking kit and then anti-mouse IgG to completely block the first primary antibodies. The sections were then incubated with the NF-1G, CMS-10, or TH antibodies at 4 °C overnight. Each section was treated with a biotinylated anti-mouse IgG followed by treatment with Rhodamine Avidin D (Vector Laboratories, USA). The exposure parameters for confocal laser scanning were the same across the control and infected samples, and photographic documentation was performed using a confocal laser scanning microscope (LSM 510, Carl Zeiss, Germany). Image analysis was performed using LSM Image Browser 3,5,0,223 software (Carl Zeiss, Germany).

### Immuno-gold Labeling and Transmission Electron Microscopy

When clinical signs were evident in the infected hamsters, both infected and controls were anesthetized with 16.5 % urethane and transcardially perfused with 0.1 M PBS (pH 7.4) containing 4 % paraformaldehyde (PFA) and 2.5 % glutaraldehyde (GA). After the brains were removed, the thalamic and hypothalamic regions were immediately trimmed into small pieces and kept in the fixative buffer (0.1 M PBS containing 4 % PFA and 2.5 % GA) for 2 h at 4 °C. Post-fixation was conducted in 0.1 M PBS containing 1 % osmium tetroxide, followed by dehydration with a graded ethanol series and embedding with an Embed 812 kit (EMS, USA). Ultra-thin sections (75 nm) prepared with an ultramicrotome (RMC MTXL, USA) and a nickel grid were incubated in a target retrieval solution (TRS, pH 9.0, DAKO, Denmark) for 15 min at 110 °C to completely unblock the epitopes. The sections were blocked with a blocking buffer (0.5 % BSA, 0.5 M NaCl, 0.1 % gelatin, and 0.05 % Tween-20 in PBS) and immunogold-labeled twice with 3F4 anti-PrP IgG and the R3 anti-AGEs antibody, or the 78295 anti-PrP antibody and NF-1G anti-CML IgG, or the R3 anti-AGEs antibody and NF-1G anti-CML IgG. Gold-conjugated anti-mouse IgG (10 nm) or 15 nm of gold-conjugated anti-rabbit IgG (Aurion, The Netherlands) was used as the secondary antibody. When the distance between a 10-nm gold particle and a 15-nm one is less than 15 nm, it was regarded that two gold particles co-localized in an area. Between each step, the sections were washed with the blocking buffer. The sections were counterstained with uranyl acetate and observed using transmission electron microscopy (TEM) (JEM-1011, JEOL, Japan).

### Isolation of the PrP^Sc^-Enriched Insoluble Fraction

A PrP^Sc^-enriched insoluble fraction was isolated as previously described [27, 31]. Briefly, the control and infected brains were homogenized in Tris-buffered saline (TBS, pH 7.4) containing 10 % *N*-lauroyl sarcosine in the presence of DNase І and centrifuged at 17,000 rpm for 30 min at 4 °C. The supernatant was ultracentrifuged at 150,000 *g* for 2 h at 4 °C, and the resulting pellet (PU^1st^, pellet following the first ultracentrifugation) was sonicated and resuspended in TBS (pH 7.4) containing 10 % NaCl and 0.1 % myristyl sulfobetaine (SB3-14) followed by layering onto TBS (pH 7.4) containing 10 % NaCl, 0.1 % SB3-14, and 20 % sucrose (sucrose cushion) and ultracentrifugation in the same condition. The resulting pellet (PU^2nd^, pellet following the second ultracentrifugation) was sonicated and re-suspended in TBS (pH 7.4) containing 0.1 % SB3-14 prior to treatment with PK (25 μg/insoluble fraction extracted from 1 g of brain, 2 h at 37 °C). After the PK-treated sample was ultracentrifuged in the same condition using a sucrose cushion, the resulting pellet (PU^3rd^, pellet following the third ultracentrifugation) was sonicated and re-suspended in TBS (pH 7.4) containing 0.1 % SB3-14. The post-mitochondrial (supernatant) fraction of the control preparation in which the control brains had been homogenized in TBS (pH 7.4) followed by centrifugation at 15,000 rpm for 30 min was used as the PrP^C^-containing fraction.

### Immunoprecipitation

The insoluble fraction (15 μg of total proteins) isolated from the brains of control or 263K prion-infected hamsters and 30 μg of the PrP^C^-containing post-mitochondrial fraction proteins prepared from control brains were boiled to unblock the epitopes and immunoprecipitated with NF-1G anti-CML IgG, 3F4 anti-PrP IgG, or the R3 anti-AGEs antibody. Each antibody was first coated to the surface of magnetic Dynabeads® M-280 Tosylactivated (Life Technologies, USA) according to the procedure described by the manufacturer. The antigen–antibody–magnetic bead complexes were washed several times with 0.05 % PBST using a magnet (Dynal MPC, Life Technologies, USA) and subsequently eluted by boiling in a sample-loading buffer. For immunoprecipitation, the PrP^Sc^-enriched insoluble fraction (2 μg of total proteins) isolated from the brains of 263K prion-infected animals was used as a positive control.

### Gel Staining and Western Blot

The control and 263K prion-infected brains were homogenized with 20 mM HEPES-KOH (pH 7.5) containing 150 mM NaCl, 0.5 % sodium deoxycholate, 0.1 % SDS, and protease inhibitor cocktail, followed by centrifugation at 12,000 rpm for 10 min. Then, the supernatant was used as a HEPES-soluble homogenate fraction. The insoluble fraction (2 μg of total proteins) isolated from the brains of the control or 263K prion-infected animals or the immunoprecipitates were separated using 12 % sodium dodecyl sulfate-polyacrylamide gel electrophoresis (SDS-PAGE) and stained with Coomassie Brilliant Blue (CBB) G-250 or transferred to a nitrocellulose membrane. The membrane was blocked with 5 % skim milk in 0.05 % TBST (20 mM Tris–HCl, pH 7.5, 150 mM NaCl, and 0.05 % Tween-20) for 1 h at room temperature and then incubated with mouse monoclonal NF-1G anti-CML IgG (10 μg) in PBS, or rabbit polyclonal R3 anti-AGEs antibody (1:1,000), or rabbit polyclonal 78295 anti-PrP antibody (1:5,000), or mouse monoclonal 3F4 anti-PrP IgG (1:20,000), or mouse monoclonal 3F10 anti-PrP IgG (1:30,000) in blocking solution overnight at 4 °C. The membrane was incubated with anti-mouse IgG-peroxidase or anti-rabbit IgG-peroxidase, and the antigen–antibody complexes were visualized using SuperSignal West Pico (Thermo Scientific, USA). The protein was quantified using a BCA protein assay (Thermo Scientific, USA).

### Isolation of N-Terminal AGEs from PrP^Sc^

To isolate the N-terminal AGEs from PrP^Sc^, the magnetic Dynabeads M-280 Tosylactivated coated with 3F4 anti-PrP IgG or the R3 anti-AGEs antibody were incubated with 1.0 M Tris–HCl (pH 8.5) for 48 h at room temperature to completely block nonspecific binding to the magnetic beads. The post-mitochondrial fraction (50 μg of total proteins) isolated from the control brains and the insoluble fraction (50 μg of total proteins) isolated from the 263K prion-infected brains were first incubated with the 3F4 anti-PrP IgG-coated magnetic beads. The 3F4 immune complexes were washed several times with 0.05 % PBST using a magnet. After changing the tube immediately before the last washing, the immune complexes were eluted with 0.5 M NH_4_OH containing 0.5 mM EDTA for 1 h and then neutralized with acetic acid. The PrP fractions purified from the 3F4 anti-PrP IgG-coated beads were treated with PK (50 μg/ml) for 1 h at 37 °C and then with protease inhibitor cocktail (Roche Applied Science, Germany). The PK-treated PrP fraction of the infected brains was then subjected to ultracentrifugation in which the supernatant was used for the subsequent immunoprecipitation (α in Fig. 6a and lane 5 in Fig. 6b); alternatively, the PK-treated PrP fraction was used for the subsequent immunoprecipitation (β in Fig. 6a and lanes 2 and 6 in Fig. 6b). Both fractions were incubated with R3 anti-AGEs antibody-coated magnetic beads. The R3 immune complexes were washed, eluted, and neutralized using the same buffers as described in the purification procedure to isolate PrP^Sc^. Both purified fractions were used for Dot blot or Western blot (Fig. 6).

## Results

### CML Co-localizes with PrP in Tyrosine Hydroxylase-Positive Neurons

**Fig. 1:**
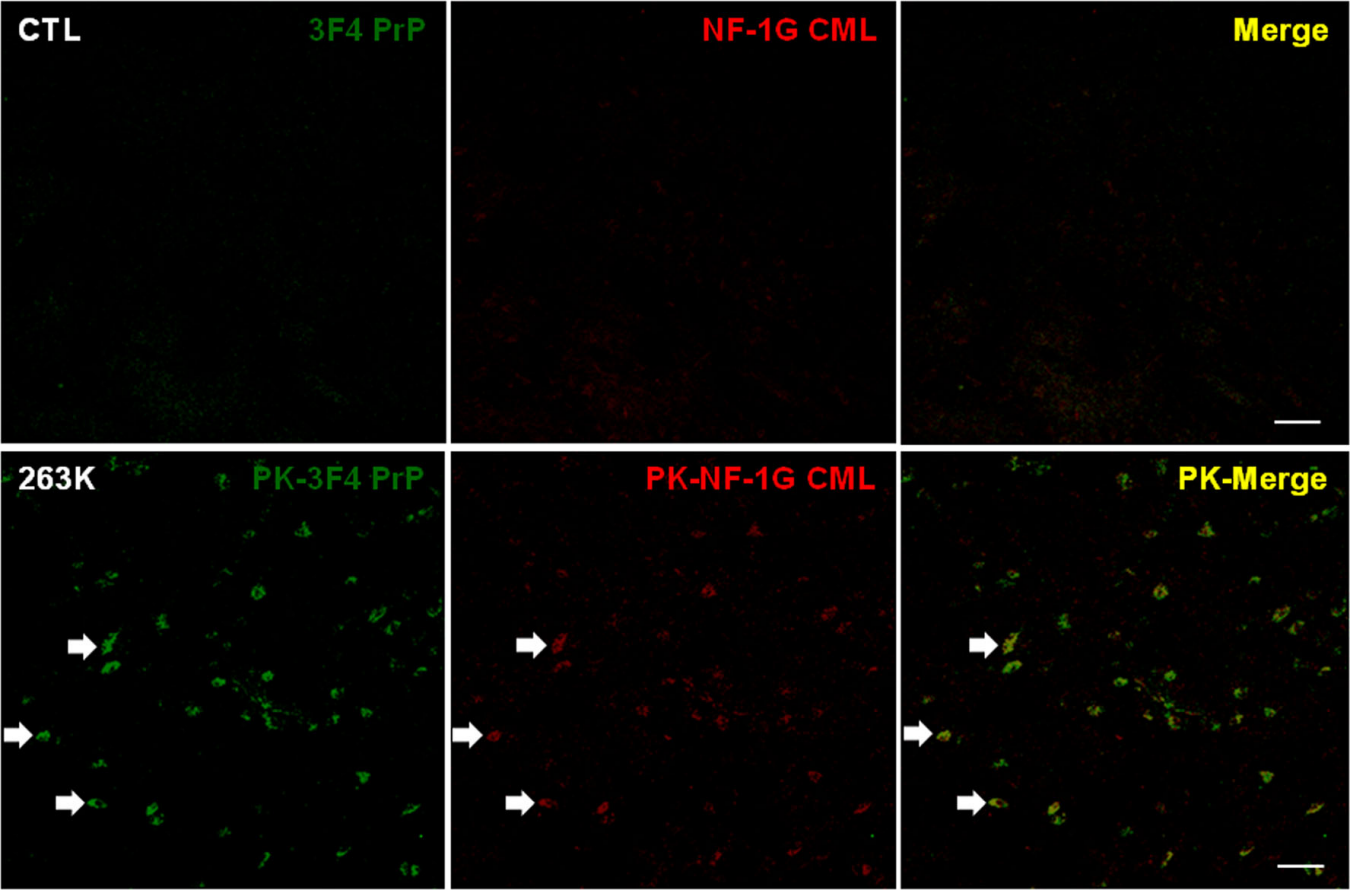
CML is localized in the PrP-positive cells within the thalamic regions of the 263K prion-infected brains. The posterior thalamic nuclear groups (po, Bregma −2.46 mm) within the thalamus of the control brains (*CTL*, *upper panels*) that were not treated with PK and the 263K prion-infected brains (*lower panels*) that were treated with PK (50 μg/ml) for 5 min at room temperature were sequentially immunostained with 3F4 anti-PrP IgG (*left panels*, *green*) and NF-1G anti-CML IgG (*central panels*, *red*), and the images were observed and merged (*right panels*, *yellow*). The *arrows* indicate the co-localization of PrP and CML. Each *bar* indicates 50 μm

**Fig. 2:**
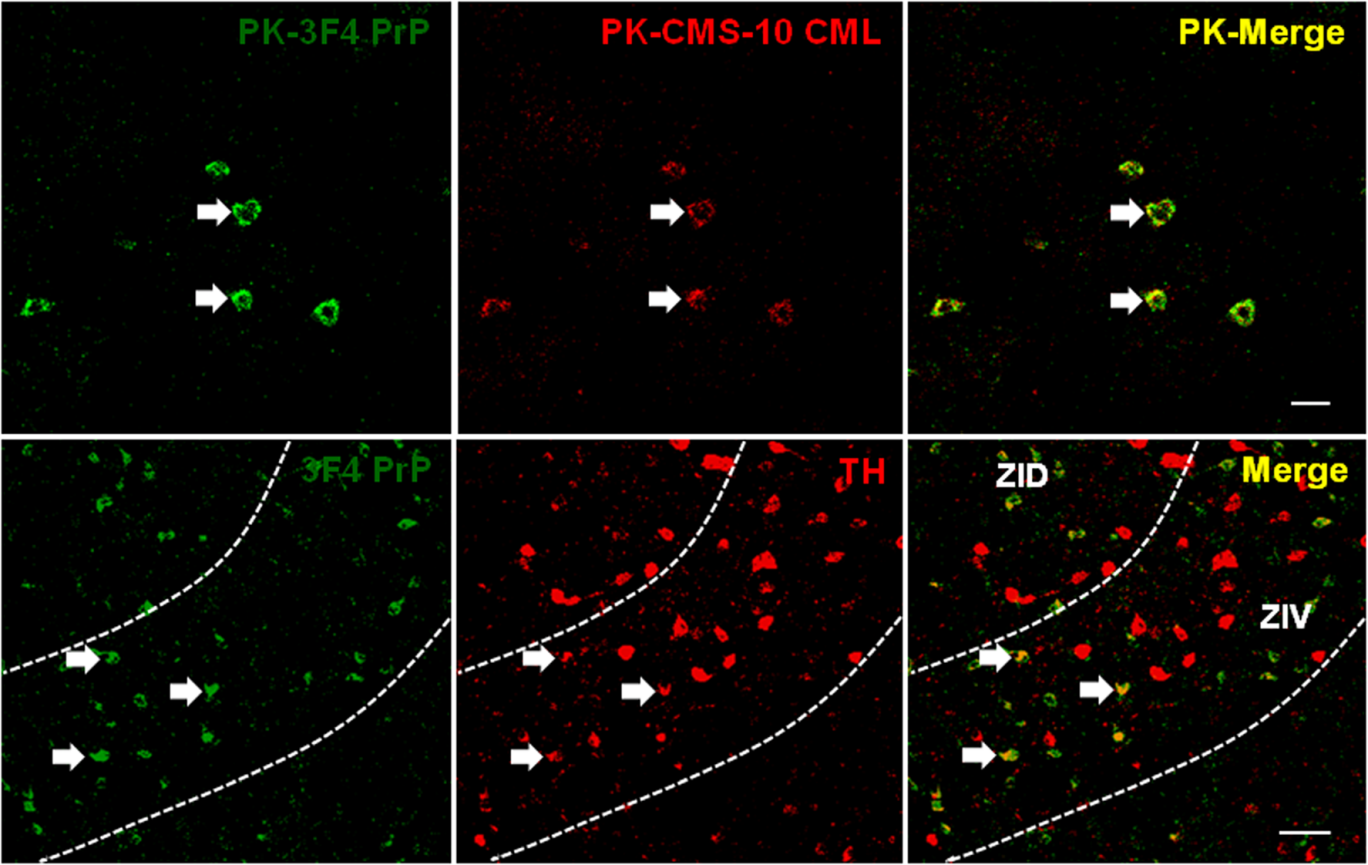
PrP is partially localized in the TH-positive neurons within the thalamic regions of the 263K prion-infected brains. The PK (50 μg/ml, 5 min, room temperature)-treated ventral posteromedial thalamic nucleus (VPM, *upper panels*, Bregma −2.46 mm) regions within the thalamus of the 263K prion-infected brains were sequentially immunostained with 3F4 anti-PrP IgG (*left upper panel*, *green*) and CMS-10 anti-CML IgG (*central upper panel*, *red*), and the images were observed and merged (*right upper panel*, *yellow*). In addition, thalamus sections from 263K prion-infected brains (“zona incerta, dorsal parts (*ZID*)” and “zona incerta, ventral parts (*ZIV*)” (*lower panels*, Bregma −2.46 mm)) were sequentially immunostained with 3F4 anti-PrP IgG (*left lower panel*, *green*) and anti-TH IgG (*central lower panel*, *red*), and the images were observed and merged (*right lower panel*, yellow). The *arrows* in the *upper* and *lower panels* indicate the co-localization of PrP and CML and of PrP and TH, respectively. The *bars* in the *right upper panel* and the *right lower panel* indicate 20 and 50 μm, respectively

**Fig. 3:**
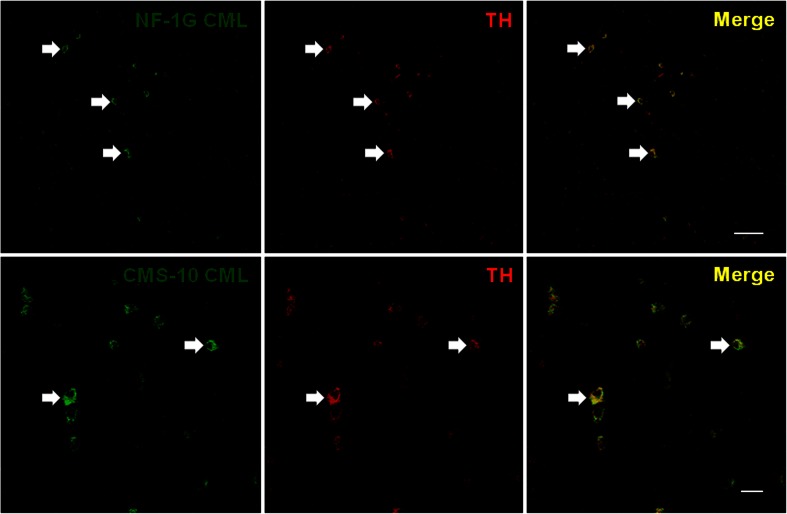
CML is localized in the TH-positive cells within the thalamic regions of the 263K prion-infected brains. Thalamus sections of 263K prion-infected brains were immunostained with NF-1G anti-CML IgG (*left upper panel*, *green*) or CMS-10 anti-CML IgG (*left lower panel*, *green*), respectively. The sections were then immunostained with anti-TH IgG (*central upper* and *lower panels*, *red*), and the images were observed and merged (*right upper* and *lower panels*, *yellow*). The *arrows* in the *upper* and *lower panels* indicate the co-localization of CML and TH. The *bars* in the *right upper panel* and the *right lower panel* indicate 50 and 20 μm, respectively. The location is Bregma −2.46 mm

We first investigated the localization of PrP and CML in control and 263K prion-infected brains. Both NF-1G IgG-positive CML and CMS-10 IgG-positive CML co-localized with the PK-resistant prion isoform in the thalamic regions of PK-treated infected brain, indicating co-localization of CML with PrP^Sc^ in the same cells (Fig. 1 and upper panels in Fig. 2). Both PrP and CML exhibited much lower abundance in the thalamic regions of control brains compared to infected brains, indicating few or no PrP and CML deposits in control brains (Fig. 1). Moreover, both PrP and CML localized within tyrosine hydroxylase (TH)-positive cells of the thalamic regions in infected brains (lower panels in Figs. 2 and 3). CML was co-localized with PK-resistant prion isoform in the parietal cortex and hippocampus of infected brains (Supplementary Fig. 1).

### CML-Positive Proteins Are Present in the PrP^Sc^-Enriched Insoluble Fraction

**Fig. 4:**
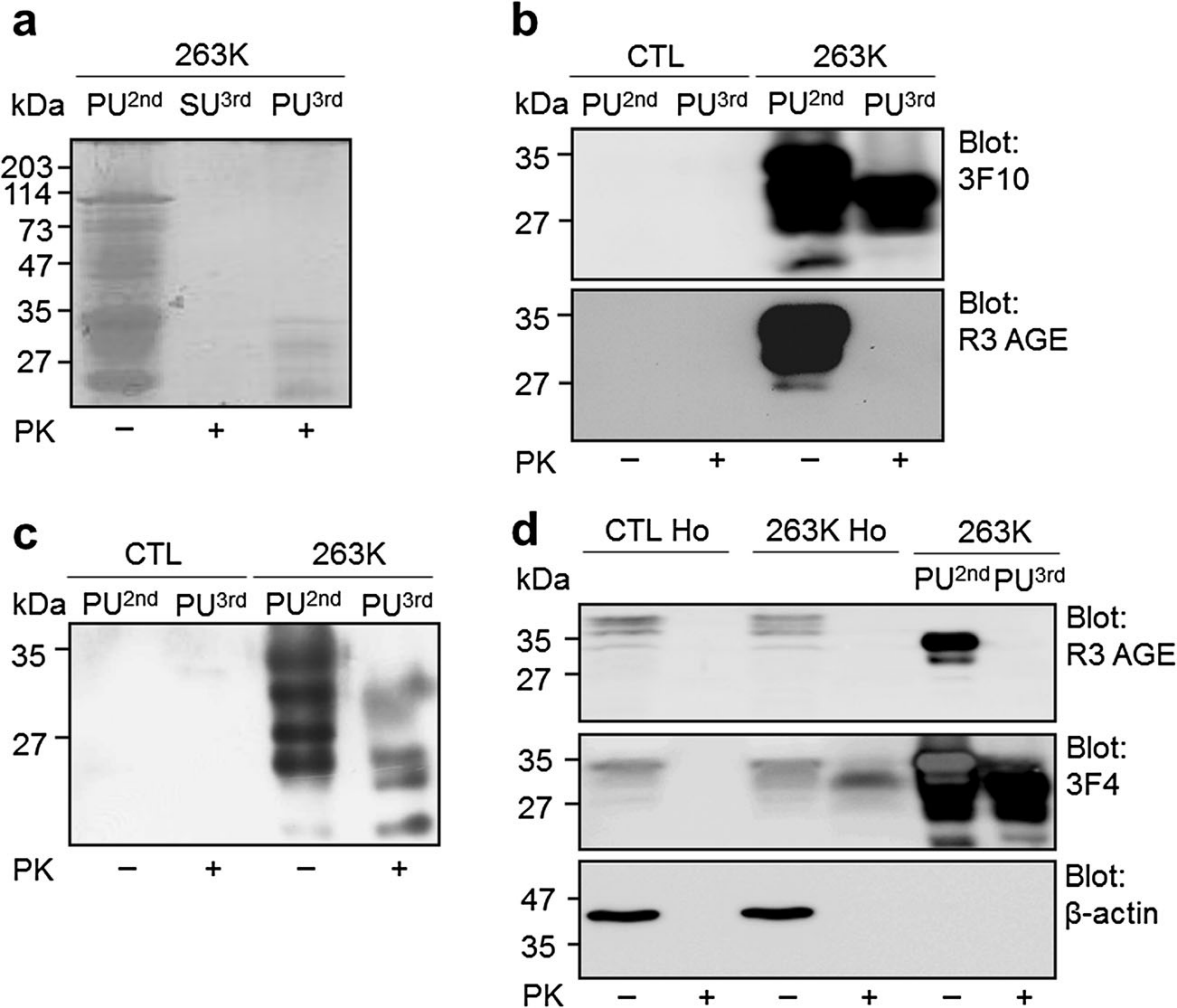
CML is linked to specific proteins in the 263K prion-infected brains. The separated proteins (15 μg of total proteins) from the insoluble pellet fractions isolated from the controls (*CTL*) or the 263K prion-infected brains were stained with CBB G-250 (**a**) or incubated with 3F10 anti-PrP IgG (*upper panel* in **b**), the R3 anti-AGEs antibody (*lower panel* in **b**), or NF-1G anti-CML IgG (**c**). In **d**, in the *two left lanes*, we applied 30 μg of total proteins in 20 mM HEPES-soluble homogenate (*Ho*) fractions of the control brains; the same quantities for 263K Ho are in the *middle lanes*. In the *two right lanes*, 1.0 μg of total proteins of the non-digested insoluble fraction (PU^2nd^) and 0.1 μg of total proteins of the digested insoluble fraction (PU^3rd^) were separated and then blotted with the R3 anti-AGEs antibody (*upper panel* in **d**). The R3 anti-AGE antibody was stripped, and the membrane was reprobed with 3F4 anti-PrP IgG (*central panel* in **d**) and subsequently reprobed with anti-β-actin IgG (*lower panel* in **d**). The PrP^Sc^-enriched insoluble pellet fractions (PU^2nd^) following the second ultracentrifugation using a sucrose cushion were digested with PK (+), followed by the third ultracentrifugation. Following the third ultracentrifugation, SU^3rd^ and PU^3rd^ indicate the supernatant and the PK-digested PrP^Sc^-enriched insoluble pellet fractions, respectively. In **d**, 30 μg of total proteins in the HEPES-soluble fractions of the controls or the infected brains was digested with 0.3 μg of PK for 1 h at 37 °C. The molecular weights (kDa) are shown on the *left side* of each figure

We next determined whether CML was associated with the PrP^Sc^ deposits found in infected brains. Initially, PrP^Sc^-enriched insoluble fraction from infected brains was isolated using ultracentrifugation (Fig. 4a). The PrP^Sc^-enriched insoluble fraction (PU^2nd^) was isolated from the infected brains using ultracentrifugation with a sucrose cushion. A portion of the PrP^Sc^-enriched insoluble fraction was treated with PK, followed by an additional ultracentrifugation using a sucrose cushion and yielding supernatant SU^3rd^ and pellet PU^3rd^. The proteins in the three fractions were separated and stained with Coomassie Brilliant Blue G 250. As shown in Fig. 4a, the PrP^Sc^-enriched insoluble fraction isolated from the infected brains (PU^2nd^) contained numerous proteins, including several proteins that likely represent PrP^Sc^ isoforms with molecular weights of less than 35 kDa; however, although several PK-resistant PrP^Sc^ isoforms were present in the PK-treated insoluble fraction following the limited proteolysis (PU^3rd^), they were not present in the supernatant (SU^3rd^). Next, we identified the full-length and truncated PrP^Sc^ isoforms in the PU^2nd^ and PU^3rd^ fractions. We identified three R3 anti-AGEs antibody-positive proteins in the PU^2nd^ fraction (Fig. 4b), as observed in a previous report [27]. PrP positivity was not observed in the insoluble fraction isolated from control brain, indicating that normal PrP^C^ was not precipitated by the ultracentrifugation (Fig. 4b). We next identified whether CML positivity was present in the PrP^Sc^-enriched insoluble fraction. Using NF-1G anti-CML IgG, we found five proteins containing CML adduct on their Lys residue(s) in the insoluble fraction isolated from infected brains but not in the insoluble fraction from the control brains (the first through the third lanes from the left in Fig. 4c). Four CML-linked proteins were identified even in the PK-digested insoluble fraction (PU^3rd^) of infected brains. These proteins exhibited apparent molecular masses that were nearly identical to those of the truncated PrP^Sc^ isoforms following PK proteolysis in SDS-PAGE analysis (the first lane from the right in Fig. 4c). The three R3-positive AGEs-linked proteins were only detected in the insoluble fraction that was not digested with PK (Fig. 4b, d). In contrast, no differential AGE positivity was seen in soluble fractions (Ho) of control and infected brains (Fig. 4d).

### Pathogenic Prion Isoforms Are Modified by CML at One or More Lys Residues

**Fig. 5:**
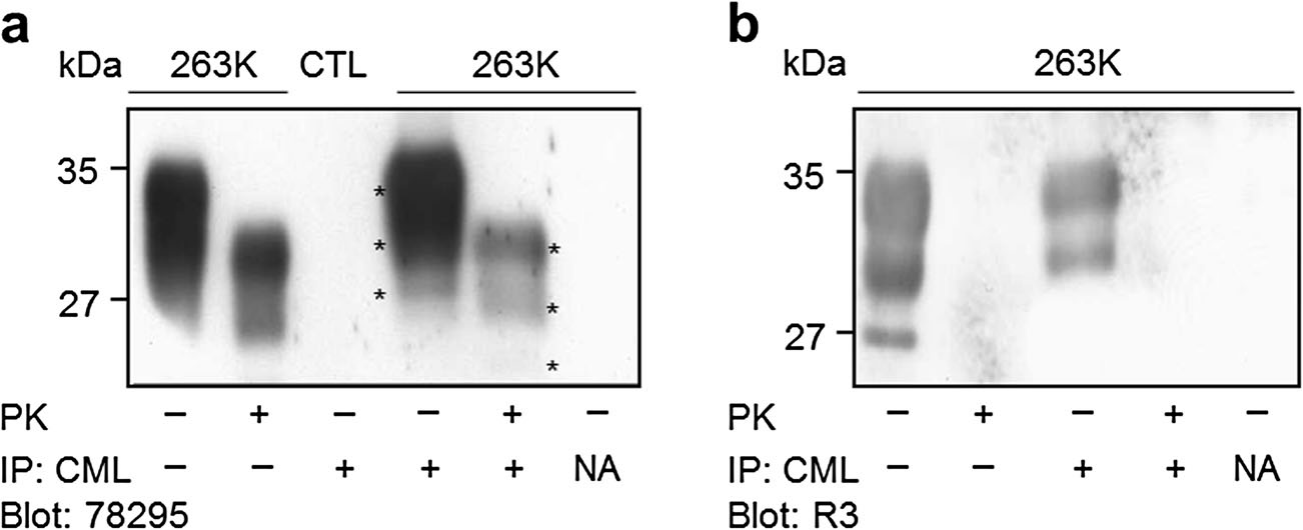
N^ε^-carboxymethyl is linked to at least one Lys residue in each of three glycosylated types of disease-associated PrP^Sc^ isoforms in 263K prion-infected brains. The insoluble fractions isolated from the controls (*CTL*) or the 263K prion-infected brains and digested with PK (+) were immunoprecipitated (*IP*) with NF-1G anti-CML IgG (*lanes 3*, *4*, and *5* in **a** and *lanes 3* and *4* in panel **b**) and blotted with the 78295 anti-PrP antibody (**a**) or R3 anti-AGEs antibody (**b**) *NA* no antibody. Magnetic beads that had not been coated with antibodies and only blocked with Tris were incubated with the insoluble fractions of the infected brains (*lane 6* in **a** and *lane 5* in **b**). The *asterisks* in **a** indicate three glycosylated (di-, mono-, and non-) PrP isoforms. The molecular weights (kDa) are shown on the *left side* of each figure

Next, we ascertained whether the pathogenic prion isoform is modified with CML. An immunoprecipitation analysis using NF-1G anti-CML IgG and the 78295 anti-PrP antibody identified CML-positive protein associated with each PrP^Sc^ isoform (Fig. 5a). Therefore, at least one Lys residue in the PK-resistant core region of the PrP^Sc^ isoform was modified with CML. No such effect was observed in control brains. Moreover, in Fig. 5b, we observed that the CML-positive immune complexes that were immunoprecipitated by NF-1G anti-CML IgG using the non-PK-treated insoluble fraction of the infected brains were detected by the R3 anti-AGEs antibody (the first and the third lanes from the left in Fig. 5b). Immune complexes were not seen using the PK-treated insoluble fractions (the second and the fourth lanes from the left in Fig. 5b). No positivity was observed for immunoprecipitation without an antibody (NA), excluding the possibility that PrP^Sc^ is nonspecifically bound to the magnetic beads (Fig. 5a, b, first lane from the right, respectively). The immunoprecipitation analyses indicated that at least one Lys residue in each of the three glycosylated (di-, mono-, and non-) types of the pathogenic prion isoforms was modified with CML.

### CML Is an N-Terminal AGE of PrP^Sc^

**Fig. 6:**
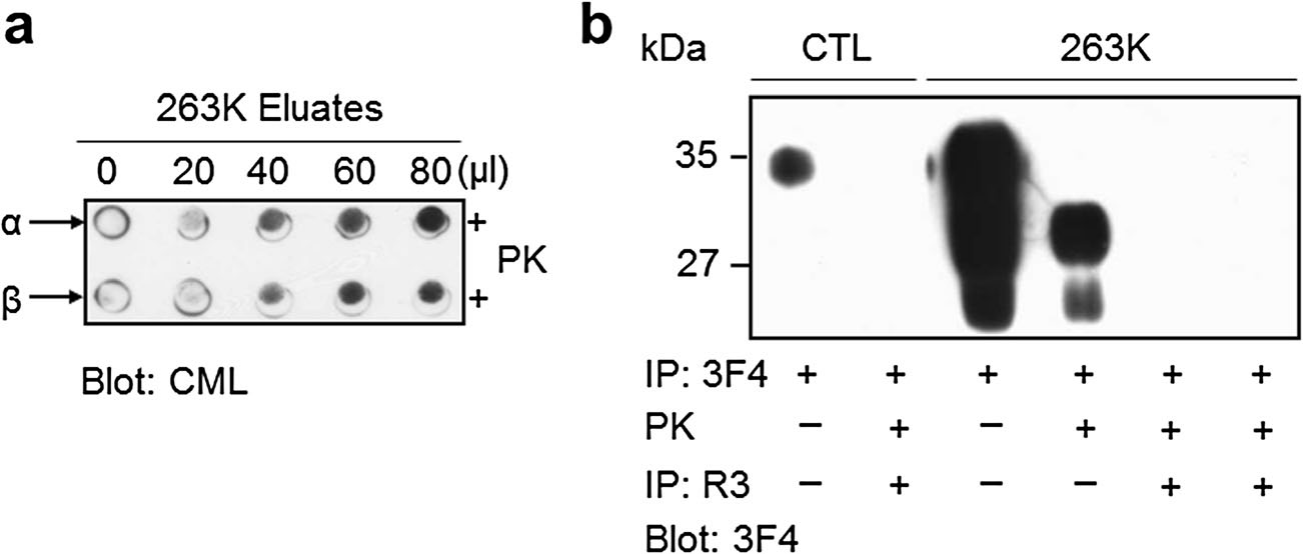
CML is the N-terminal AGE at the PK-sensitive region of disease-associated PrP^Sc^ isoforms. The post-mitochondrial fractions of the controls (*CTL*) and the insoluble fractions of the 263K prion-infected brains were immunoprecipitated (*IP*) with 3F4 anti-PrP IgG. The PrP fractions that had been eluted from the 3F4 immune-complexes were treated with PK and protease inhibitor cocktail and were subjected to ultracentrifugation, in which the supernatant was used for the subsequent immunoprecipitation with the R3 anti-AGE antibody (*α* in **a** and *lane 5* in **b**), or the PK-digested PrP fraction was used for the subsequent IP with the R3 anti-AGEs antibody without ultracentrifugation (*β* in **a** and *lanes 2* and *6* in **b**). Both fractions (*α* and *β* in **a** and *lanes 2*, *5*, and *6* in **b**) were then incubated with R3 anti-AGEs antibody-coated magnetic beads. The eluates from the R3 immune-complexes were subjected to Dot-blotting with NF-1G anti-CML IgG (**a**) or Western-blotting with 3F4 anti-PrP IgG (**b**). *Lanes 5* and *6* in **b** received 40 μl of each eluate, whereas *lanes 1* through *4* got 10 μl

We previously showed that AGEs are linked to PrP^Sc^ at one or more Lys residues at 23, 24, and 27 in the N-terminal region of PrP^Sc^ [27]. To clarify whether the N-terminal AGEs of PrP^Sc^ are CMLs, we affinity-purified the R3 anti-AGEs antibody-positive species that were immunoprecipitated by 3F4. Subsequently, these immunoprecipitates were eluted from the supernatant fraction, in which soluble forms of proteins and the truncated non-insoluble amino acid residues by PK proteolysis reside, after PK digestion and ultracentrifugation (Fig. 6a, α) or from the PK-treated PrP fraction (Fig. 6a, β) following the purification of PrP^Sc^ from the insoluble fraction with 3F4-coated magnetic beads. CML immunoreactivity was increased for both of the eluates sequentially purified from the 3F4-beads and the R3-bead immune complexes in a loaded volume-dependent manner (Fig. 6a). No prion isoforms were found in either of the eluates purified by 3F4 and R3 antibodies (the fifth and the sixth lanes from the left in Fig. 6b), whereas the prion isoforms were detected in the eluates from the 3F4-bead immune complexes used as positive controls (the third and the fourth lanes from the left in Fig. 6b). These results indicate that neither of the CML-positive eluates purified by the 3F4 and R3 antibodies contained the proteolytically truncated PrP^Sc^; rather, they contained at least one CML-linked Lys residue or a CML-linked peptide fragment(s) proteolytically dissociated from the N-terminus of PrP^Sc^ due to PK treatment that digests the N-terminal region (23–89) of PrP^Sc^ in 263K-infected hamster whereas leaves the truncated PK-resistant PrP^Sc^ isoform (PrP 90–231) [1]. In addition, nonspecific binding of the proteolytically truncated PrP^Sc^ to the R3 anti-AGEs antibody-coated magnetic beads was not observed (the first lane from right in Fig. 6b). These findings indicate that at least one of the three Lys residues at positions 23, 24, and 27 at the N-terminus of PrP^Sc^ was linked with CML. Alternatively, at least one Lys residue might be involved in the formation of other complex AGE structures containing a CML moiety.

### CML-Linked PrP^Sc^ Localizes in the Cellular Compartments of Neurons

**Fig. 7:**
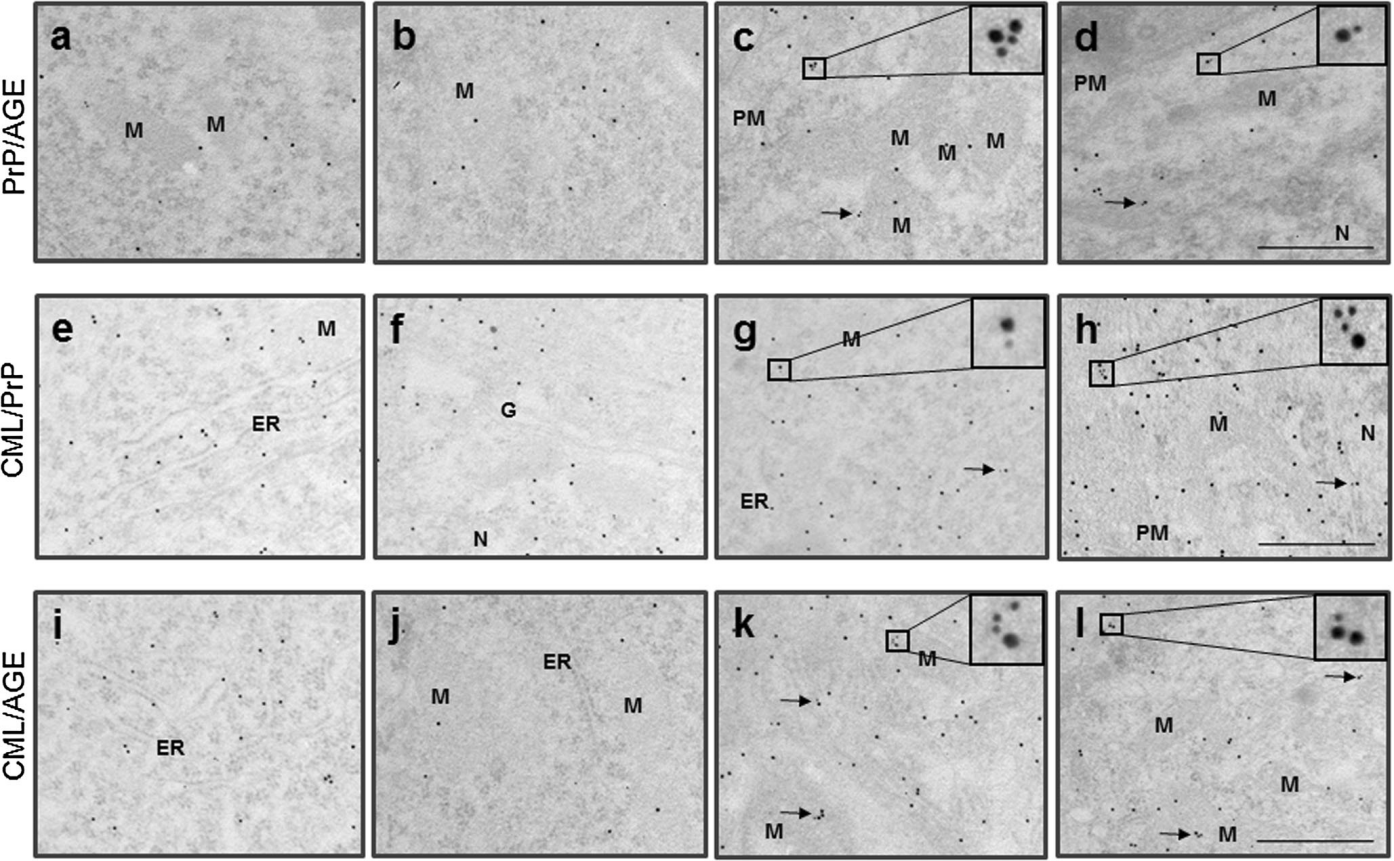
The prion isoform is co-localized with CML (or AGEs) not only in the plasma membrane but also in the intracellular compartments of the thalamic and hypothalamic nuclei in 263K prion-infected brains. The controls (**a**, **b**, **e**, **f**, **i**, and **j**) and 263K prion-infected (**c**, **d**, **g**, **h**, **k**, and **l**) brain sections were immunogold-labeled twice with AGEs (R3, 15-nm gold particles) and PrP (3F4, 10-nm gold particles) (**a**–**d**), PrP (78295, 15 nm), and CML (NF-1G, 10 nm) (**e**–**h**) or AGEs (R3, 15 nm) and CML (NF-1G, 10 nm) (**i**–**l**) in the thalamic (**a**, **c**, **e**, **g**, **i**, and **k**) and hypothalamic regions (**b**, **d**, **f**, **h**, **j**, and **l**) and observed by TEM. Note the co-localization (*arrows* and *square boxes*) of AGEs- (R3, 15 nm) and PrP-positivities (3F4, 10 nm) in the plasma membrane (*PM*), mitochondria (*M*), and cytosol in **c** and **d**, PrP- (78295, 15 nm) and CML-positivities (NF-1G, 10 nm) in the nuclear (*N*) membrane and cytosol in **g** and **h**, and AGEs- (R3, 15 nm) and CML-positivities (NF-1G, 10 nm) in the mitochondria (*M*) and cytosol in **k** and **l** within the thalamic and hypothalamic nuclei of the infected brain. *Scale bars* represent 500 nm

Next, we performed a TEM analysis to identify the localization of CML-linked PrP^Sc^ within cells of the infected brain (Fig. 7). The co-localization of PrP and AGEs was observed in the plasma membrane, mitochondria, and cytosol within the thalamic and hypothalamic nuclei of the infected brains, whereas no co-localization was observed in the control brains (Fig. 7a–d). PrP co-localized with CML in the nuclear membrane and in the cytosol within the thalamic and hypothalamic nuclei of the infected brains but not in these regions of control brains (Fig. 7e–h). In addition, AGEs co-localized with CML in the mitochondria and cytosol within the thalamic and hypothalamic nuclei of the infected brains but not those of the control brains (Fig. 7i–l). These observations indicate that both glycophosphatidylinositol-anchored and intracellular PrP isoforms (which are likely to be PrP^Sc^) were modified with CML in the plasma membrane and in specific intracellular regions of the thalamic and hypothalamic nuclei.

## Discussion

In this study, we observed that CML and the prion isoform (PrP^Sc^) were deposited in numerous brain areas (data not shown), including the parietal cortex and hippocampus in 263K prion-infected hamsters; however, few or no deposits of CML or PrP were observed in the controls. CML was shown to be extensively co-localized with PrP^Sc^ in the thalamic regions of the infected brains, and the CML was linked to at least one Lys residue at the N-terminus of PrP^Sc^ as well as to at least one of eight Lys residues on the PK-resistant core region of PrP^Sc^. The CML linkage to PrP^Sc^ (i.e., the modification of PrP^Sc^ with CML) occurred in the TH-positive neurons within the thalamic regions of infected brains. The current study found that TH, which is the rate-limiting enzyme of dopamine biosynthesis in the dopaminergic neurons of the basal ganglia and nigrostriatal system, was localized in numerous thalamic nuclei of the infected brains; previous studies have reported similar results [32–34]. Thus, we assume that both the prion isoform- and the CML-positive cells would be TH-positive dopamine-like neurons in the thalamic regions of the infected brains. Although numerous PrP-positive cells were TH-positive in most of the thalamic regions of the infected brains (data not shown), certain 3F4-positive PrP isoforms were only localized in a number of TH-positive neurons, particularly in the “zona incerta, dorsal parts” and the “zona incerta, ventral parts” of the thalamic region in infected brains (but not in all TH-positive neurons; see Fig. 2). Although the infected brains were harvested at the terminal stage, not all TH-positive neurons in the thalamic region of the infected brains expressed PrP isoforms (see Fig. 2). In addition, the PrP isoform within certain TH-positive neurons in the infected brains must have been PrP^Sc^ constituting prions because the normal prion isoform (PrP^C^) was expressed at basal levels. These neurons were observed less often in the control brains (see Fig. 1). These observations indicate that prion conversion occurred in numerous, but not all, TH-positive dopamine-like neurons within the thalamic regions of the infected brains, suggesting the co-localization of the prion protein and TH since a recent study has reported an interaction between the prion protein and TH [35].

The NF-1G anti-CML IgG used in this study detected several CML-positive proteins with molecular weights of less than 27 kDa, whereas the R3 and 6D12, anti-AGEs antibodies, which were used in a previous study, did not (Fig. 4c) [27]. Our finding indicates that the specific PrP^Sc^ isoforms with molecular weights of less than 27 kDa in infected brains may be modified with CML. In a previous study, R3 detected three AGEs-modified pathogenic prion isoforms in the non-PK-treated PrP^Sc^-enriched insoluble fraction at the positions identical to di-, mono-, and non-glycosylated prion isoforms, whereas 6D12 detected two AGE-modified pathogenic prion isoforms at the positions identical to di- and mono-glycosylated prion isoforms [27]. However, neither R3 nor 6D12 anti-AGEs antibodies were able to detect any isoform of the pathogenic prion protein in the PK-treated insoluble fraction, indicating that both anti-AGEs antibodies detected AGE structures located only at the PK-sensitive N-terminal portion of the pathogenic prion isoform. The CML-positive proteins, which appear as truncated PrP^Sc^ isoforms after PK proteolysis, were detected in the insoluble fraction isolated from the infected brains even after PK proteolysis (see Fig. 4c). This observation indicates a linkage between N^ε^-carboxymethyl and at least one of the eight Lys residues on the PK-resistant core region of PrP^Sc^ from infected hamster brain. Eight Lys residues are present in the PK-resistant core region of the golden hamster prion protein at positions 101, 104, 106, 110, 185, 194, 204, and 220 (P04273 in UniprotKB).

The result shown in Fig. 5b suggests the following regarding the position of the N^ε^-carboxymethyl modification of PrP^Sc^ isoforms in the infected brains: One or more PK-sensitive N-terminal CML moieties on the PrP^Sc^ isoform were immunoprecipitated and then positively immunoreacted with the R3 anti-AGEs antibody. This observation suggests that N^ε^-carboxymethyl is linked to the side chain of at least one PK-sensitive N-terminal Lys residue. Additionally, the specific PrP^Sc^ isoform modified with CML at one or more Lys residues in the PK-resistant core region may also be modified with AGEs (i.e., CML) at its N-terminus. Notably, the R3 anti-AGEs antibody does not detect the CML moiety on the PK-resistant core of the specific PrP^Sc^ but can detect CML moieties in AGE structure(s) on the N-terminus. This finding suggests the possible following scenarios: the CML structure is linked at the PK-sensitive N-terminus of specific PrP^Sc^ isoforms; however, this “CML” might actually be a CML-like structure(s) containing a CML moiety, which differs slightly from the structure of CML. The prion protein contains two consecutive Lys residues at positions 23 and 24 in the PK-sensitive N-terminus that may be linked to a more complex AGE structure containing a CML moiety via nonenzymatic glycation of the individual amino groups of side chains. Thus, the R3 anti-AGEs antibody may detect a more complex CML moiety-containing AGE structure on the side chains of Lys 23 and Lys 24, as well as CML on Lys 27. This interpretation suggests that the N-terminal AGE structure of PrP^Sc^ is a CML or a more complex structure containing a CML moiety produced by glycation of the consecutive Lys residues’ side amino groups including the N-terminal amine of Lys 23; this structure may be similar to the methylglyoxal-lysine dimer or glyoxallysine dimer, which are Lys–Lys cross-linked AGEs [36]. As suggested, the lack of an experimentally determined structure of PrP^Sc^ may be due to the variants of PrP^Sc^ that undergo kaleidoscopic changes in in vivo cells or tissues [3]. Thus, the CML-modified PrP^Sc^ isoforms may represent a variant of PrP^Sc^. More elaborate techniques must be developed to isolate the PK-sensitive N-terminal AGE structure of PrP^Sc^ (e.g., developing a protease[s] that specifically cleaves the peptide bond between Lys and Lys, between Lys and Arg, or between Pro and Lys in the N-terminal sequence (23KKRPKP28) of prion proteins; this N-terminal sequence is identical in nearly all mammalian species).

AGEs can be formed by a series of reactions, called nonenzymatic glycation, between amino groups of amino acids (particularly Lys or Arg) and carbohydrates in vitro and in vivo, thereby affecting the aging process and neurodegenerative disorders including Alzheimer’s disease [20, 36, 37]. A TBS–sucrose cushion buffer was used in the second and third ultracentrifugation procedures to isolate the PrP^Sc^-enriched insoluble fractions in this study. Therefore, it is necessary to determine whether the CML modification of PrP^Sc^ occurs via the glycation between PrP^Sc^ and sucrose during the in vitro ultracentrifugation procedures using a sucrose cushion. We determined that the PrP^Sc^ isoform of 263K prion-infected brains was not nonenzymatically glycated with sucrose (or glucose) via in vitro glycation of the PrP^Sc^-containing PU^1st^ (the pellet fraction isolated following the first ultracentrifugation) (unpublished data). Neither sucrose nor glucose induced AGEs modification of the pathogenic prion isoform in vitro. Therefore, the CML-linked (or AGEs-linked) prion protein was produced in infected brains. In addition, a previous study showed that the NF-1G anti-CML antibody was specific to CML that had been produced from the nonenzymatic glycation between amino acid Lys in the presence of a reducing agent (NaCNBH_3_) or from bovine serum albumin-derived AGEs in the presence of the reducing agent [38].

The current study showed that at least one Lys residue at the PK-sensitive N-terminus of PrP^Sc^ was modified with CML, and there is CML on at least one of the eight Lys residues on the PK-resistant core of PrP^Sc^. In addition, CML modification of PrP^Sc^ appeared to occur primarily in the plasma membrane, nuclear membrane, mitochondria, and cytosol of the prion-affected cells, especially in TH-expressing cells in thalamic and hypothalamic regions, parietal cortex, and hippocampus. The role(s) of the AGEs-modified pathogenic prion isoform in prion-infected brains must be elucidated further.

## Electronic supplementary material

Below is the link to the electronic supplementary material.

Supplementary Fig. 1 CML is localized in the PrP-positive cells in the parietal cortex and hippocampus of the 263K prion-infected brains. The parietal cortex (upper panels) and hippocampus (lower panels) in the 263K prion-infected brains that were treated with PK (50 μg/ml) for 5 min at room temperature were sequentially immunostained with 3F4 anti-PrP IgG (left panels, green) and NF-1G anti-CML IgG (central panels, red), and the images were observed and merged (right panels, yellow). The arrows indicate the co-localization of PrP and CML. Each bar indicates 20 μm. (GIF 316 kb)

High resolution image (TIFF 27376 kb)
