# Supplementary figures and images for: Nε-Carboxymethyl Modification of Lysine Residues in Pathogenic Prion Isoforms
Source: Mol Neurobiol. 2015 May 16;53(5):3102–12. doi: 10.1007/s12035-015-9200-8 (PMC4902843; doi:10.1007/s12035-015-9200-8)

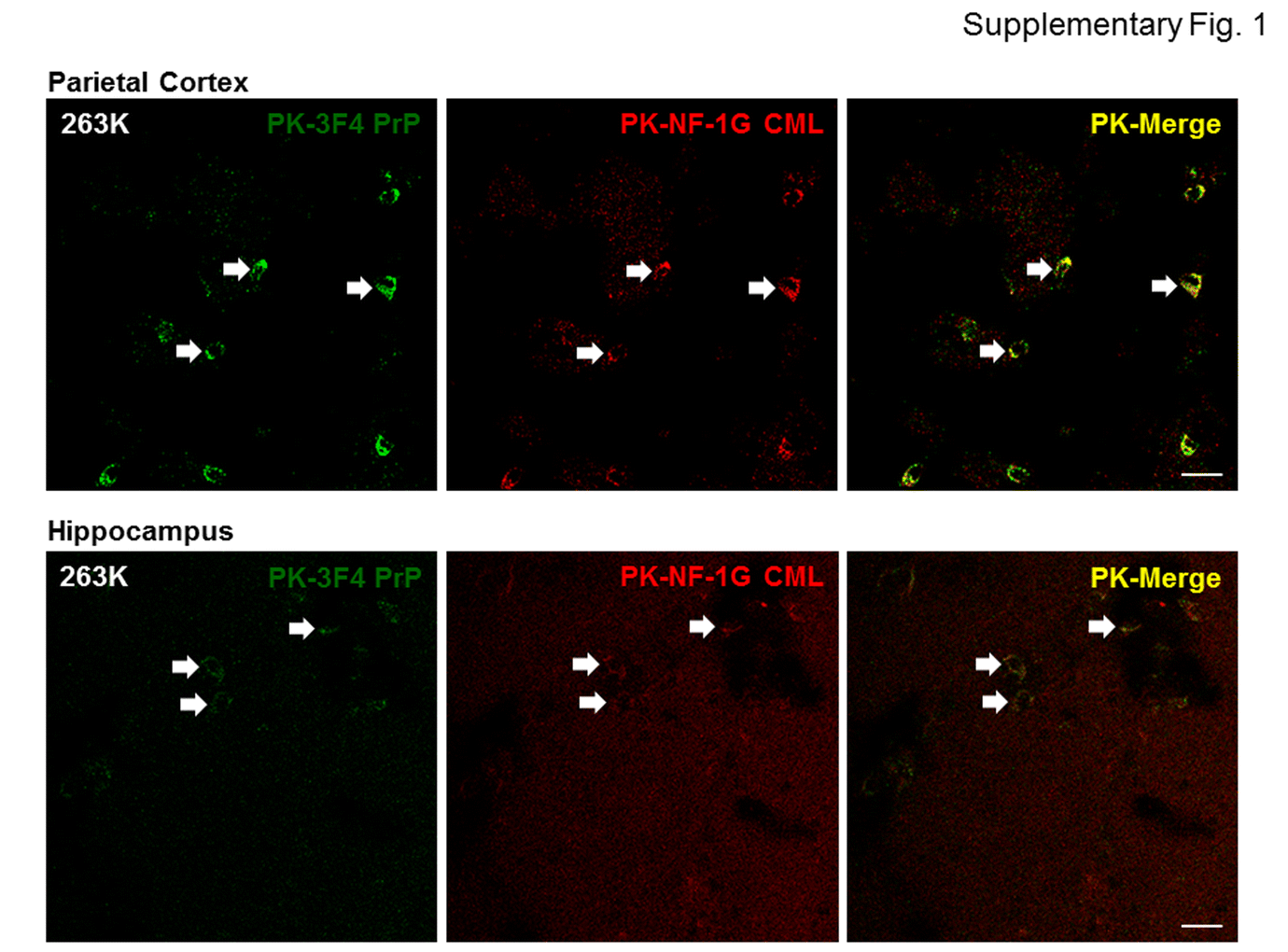

Supplement: Supplementary file 1 — CML is localized in the PrP-positive cells in the parietal cortex and hippocampus of the 263K prion-infected brains. The parietal cortex (upper panels) and hippocampus (lower panels) in the 263K prion-infected brains that were treated with PK (50 μg/ml) for 5 min at room temperature were sequentially immunostained with 3F4 anti-PrP IgG (left panels, green) and NF-1G anti-CML IgG (central panels, red), and the images were observed and merged (right panels, yellow). The arrows indicate the co-localization of PrP and CML. Each bar indicates 20 μm. (GIF 316 kb) [file 12035_2015_9200_Fig8_ESM.gif]

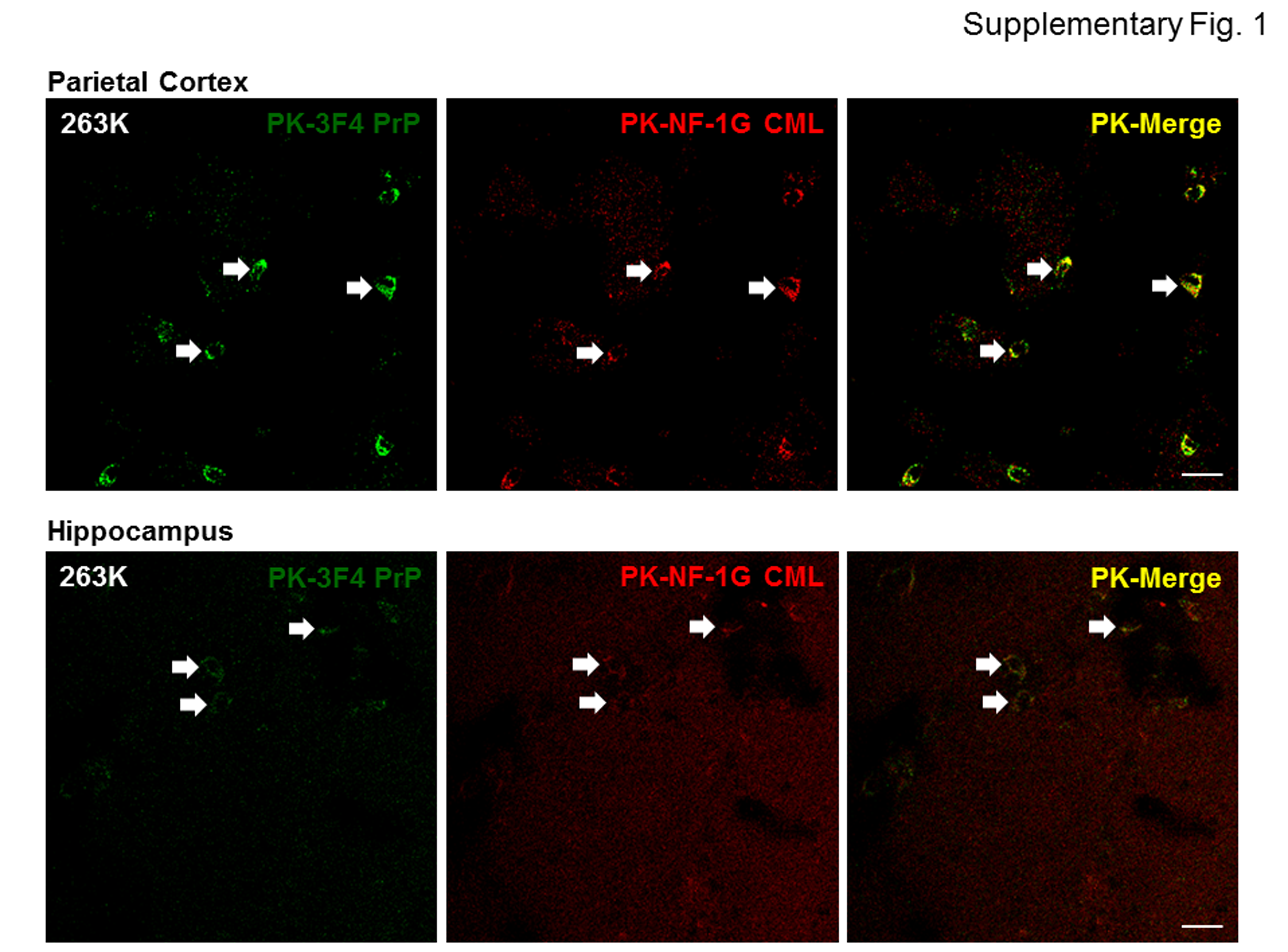

Supplement: Supplementary file 2 — High resolution image (TIFF 27376 kb) [file 12035_2015_9200_MOESM1_ESM.tif]
